# Supplementary material for: Effect of composting and storage on the microbiome and resistome of cattle manure from a commercial dairy farm in Poland
Source: Environ Sci Pollut Res Int. 2024 Apr 15;31(21):30819–35. doi: 10.1007/s11356-024-33276-z (PMC11096248; doi:10.1007/s11356-024-33276-z)
Supplement: Supplementary file 1 — Supplementary file1 (DOCX 454 KB) [file 11356_2024_33276_MOESM1_ESM.docx]

Supplementary Material

**Table S1:** Changes during manure treatments in **A:** humidity [%], **B:** pH, and temperature [°C] changes during manure treatments

**A:** Humidity [%] changes during manure treatments

| Treatment strategy | Duration [days] | | | | | | | |
| --- | --- | --- | --- | --- | --- | --- | --- | --- |
|  | 3 | 7 | 14 | 21 | 28 | 35 | 42 | 70 |
| Compost | >80 | >80 | >80 | >80 | 75 | 70 | 65 | 65 |
| Storage | >80 | >80 | >80 | >80 | 65 | 65 | 60 | 65 |

**B:** pH changes during manure treatments

| Treatment strategy | Duration [days] | | | | | | | |
| --- | --- | --- | --- | --- | --- | --- | --- | --- |
|  | 3 | 7 | 14 | 21 | 28 | 35 | 42 | 70 |
| Compost | 6.8 | 6.7 | 6.8 | 6.3 | 6.8 | 6.8 | 6.5 | 7.0 |
| Storage | 6.6 | 6.8 | 7.0 | 6.8 | 7.0 | 6.8 | 6.6 | 6.6 |

**C:** temperature [ °C] changes during manure treatments

| Treatment strategy | Duration [days] | | | | | | | |
| --- | --- | --- | --- | --- | --- | --- | --- | --- |
|  | 3 | 7 | 14 | 21 | 28 | 35 | 42 | 70 |
| Compost | 22.5 | 28.3 | 42.5 | 51.5 | 60.1 | 61.1 | 51.6 | 32.2 |
| Storage | 22.0 | 20.5 | 21.4 | 21.2 | 22.3 | 21.7 | 22.7 | 21.3 |

**Table S2.** Mean read count with standard deviation of each phylum across the treatment

| Phylum | Read count | | | | | | | | | |
| --- | --- | --- | --- | --- | --- | --- | --- | --- | --- | --- |
|  | Raw | | Composted 5W | | Composted 10W | | Stored 2M | | Stored 4M | |
|  | Mean | SD | Mean | SD | Mean | SD | Mean | SD | Mean | SD |
| *Pseudomonadota* | 3384.5 | 2673.571 | 7409 | 1524.522 | 7795.5 | 3434.418 | 4622 | 541.6438 | 7777.5 | 1289.056 |
| *Bacillota* | 21984.5 | 21933.75 | 2799 | 2586.597 | 2126.5 | 1963.636 | 2082 | 721.2489 | 2513 | 912.1677 |
| *Planctomycetota* | 46 | 22.62742 | 979.5 | 125.1579 | 1046.5 | 181.7264 | 679 | 98.99495 | 1483 | 427.0925 |
| *Actinomycetota* | 607.5 | 350.0179 | 2276.5 | 2023.033 | 2805.5 | 1892.925 | 3948 | 3341.787 | 3405 | 2586.597 |
| *Desulfobacterota* | 160 | 207.8894 | 75 | 60.81118 | 60.5 | 55.86144 | 0 | 0 | 0 | 0 |
| *Bacteroidota* | 9835 | 2940.15 | 2725.5 | 178.898 | 2943 | 721.2489 | 935.5 | 512.6524 | 1945 | 28.28427 |
| *Verrucomicrobiota* | 1422 | 640.6387 | 1502.5 | 115.2584 | 1259.5 | 53.03301 | 268 | 309.7128 | 595 | 72.12489 |
| *Chloroflexota* | 0 | 0 | 1285 | 1508.966 | 500 | 260.2153 | 209 | 137.1787 | 697.5 | 241.1234 |
| *Bdellovibrionota* | 0 | 0 | 422.5 | 185.9691 | 183.5 | 20.5061 | 166.5 | 14.84924 | 707 | 164.0488 |
| *Acidobacteriota* | 0 | 0 | 127.5 | 112.43 | 118.5 | 106.7731 | 26 | 36.76955 | 184 | 107.4802 |
| *Patescibacteria* | 105.5 | 60.10408 | 176 | 70.71068 | 178.5 | 13.43503 | 18 | 14.14214 | 26.5 | 20.5061 |
| *Myxococcota* | 0 | 0 | 723.5 | 136.4716 | 480 | 104.6518 | 495 | 260.2153 | 932 | 405.8793 |
| *Cyanobacteria* | 461.5 | 92.63099 | 164 | 38.18377 | 110 | 82.02439 | 69 | 39.59798 | 128.5 | 33.23402 |
| *Dependentiae* | 0 | 0 | 56.5 | 9.192388 | 23 | 2.828427 | 0 | 0 | 0 | 0 |
| WPS-2 | 0 | 0 | 34 | 5.656854 | 55 | 28.28427 | 0 | 0 | 0 | 0 |
| *Armatimonadota* | 0 | 0 | 126.5 | 37.47666 | 63.5 | 9.192388 | 0 | 0 | 0 | 0 |
| *Gemmatimonadota* | 0 | 0 | 122 | 144.2498 | 65.5 | 54.44722 | 35 | 9.899495 | 315 | 158.3919 |
| *Hydrogenedentes* | 0 | 0 | 74 | 2.828427 | 89 | 31.1127 | 0 | 0 | 0 | 0 |
| *Fibrobacterota* | 64 | 19.79899 | 65 | 28.28427 | 58.5 | 16.26346 | 30 | 42.42641 | 51 | 18.38478 |
| *Campylobacterota* | 67.5 | 88.38835 | 0 | 0 | 6.5 | 9.192388 | 12 | 16.97056 | 73.5 | 10.6066 |
| *Spirochaetota* | 612.5 | 28.99138 | 59.5 | 28.99138 | 14.5 | 20.5061 | 0 | 0 | 0 | 0 |
| *Euryarchaeota* | 126 | 25.45584 | 12 | 16.97056 | 0 | 0 | 3.5 | 4.949747 | 25.5 | 36.06245 |
| *Abditibacteriota* | 0 | 0 | 55.5 | 61.51829 | 18 | 14.14214 | 0 | 0 | 0 | 0 |
| *Deferribacterota* | 0 | 0 | 19 | 26.87006 | 8 | 11.31371 | 0 | 0 | 0 | 0 |
| *Elusimicrobiota* | 65 | 48.08326 | 0 | 0 | 0 | 0 | 0 | 0 | 0 | 0 |
| *Sumerlaeota* | 0 | 0 | 27 | 38.18377 | 0 | 0 | 0 | 0 | 0 | 0 |
| *Thermoplasmatota* | 7 | 9.899495 | 0 | 0 | 0 | 0 | 0 | 0 | 0 | 0 |
| **TOTAL** | **38948.5** | **na** | **21316.5** | **na** | **20009** | **na** | **13598.5** | **na** | **20859** | **na** |

**na** – not applicable

**Table S3.** The results of pairwise PERMANOVA analysis. The multi-testing adjustment is based on Benjamini-Hochberg procedure (FDR)

| Pair | F-value | R-squared | P-value | FDR |
| --- | --- | --- | --- | --- |
| Untreated vs Stored | 9.0866 | 0.694344 | 0.0666667 | 0.1 |
| Untreated vs Composted | 9.30967 | 0.699467 | 0.0666667 | 0.1 |
| Composted vs Stored | 1.93641 | 0.243991 | 0.215 | 0.215 |


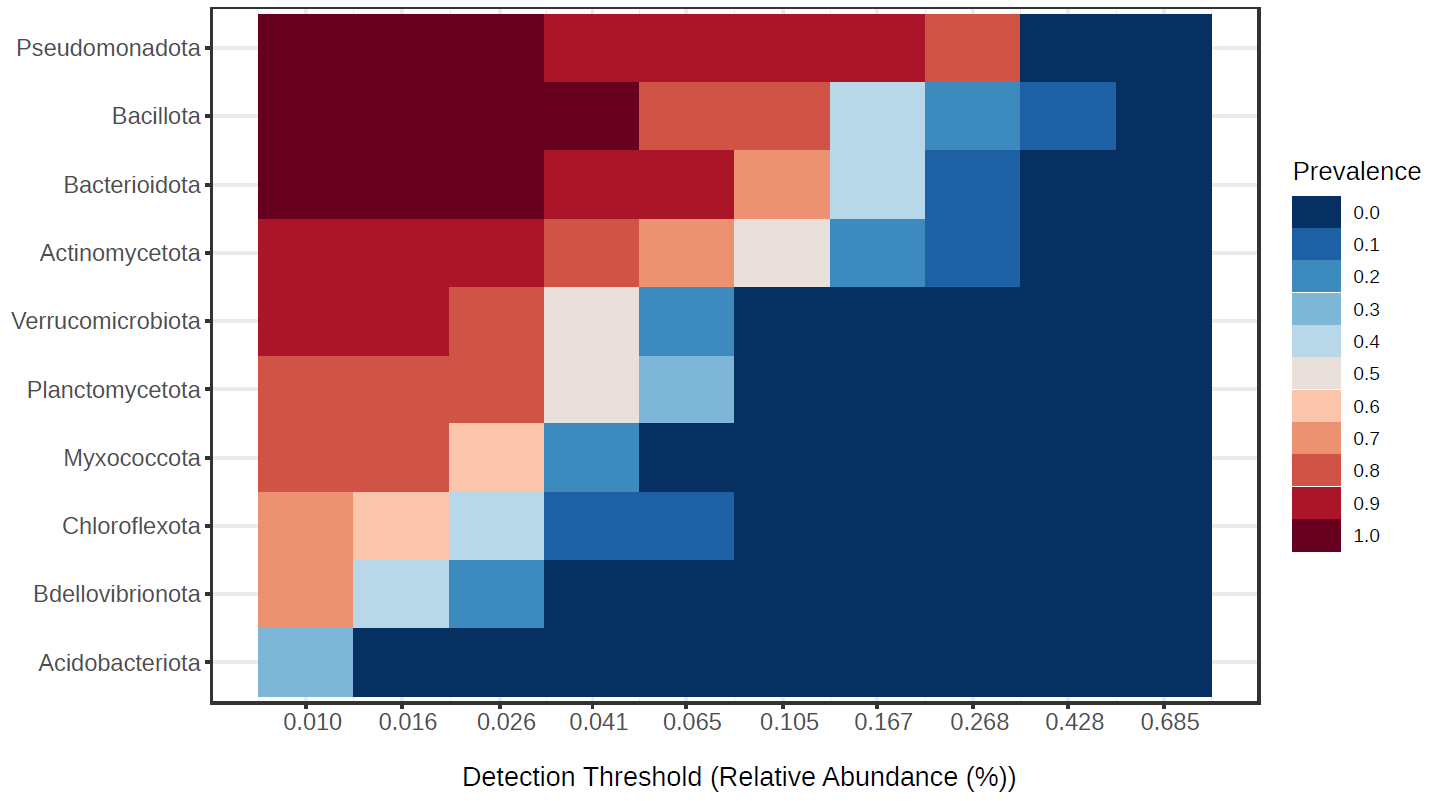


**Figure S1.** Core microbiome across analyzed samples


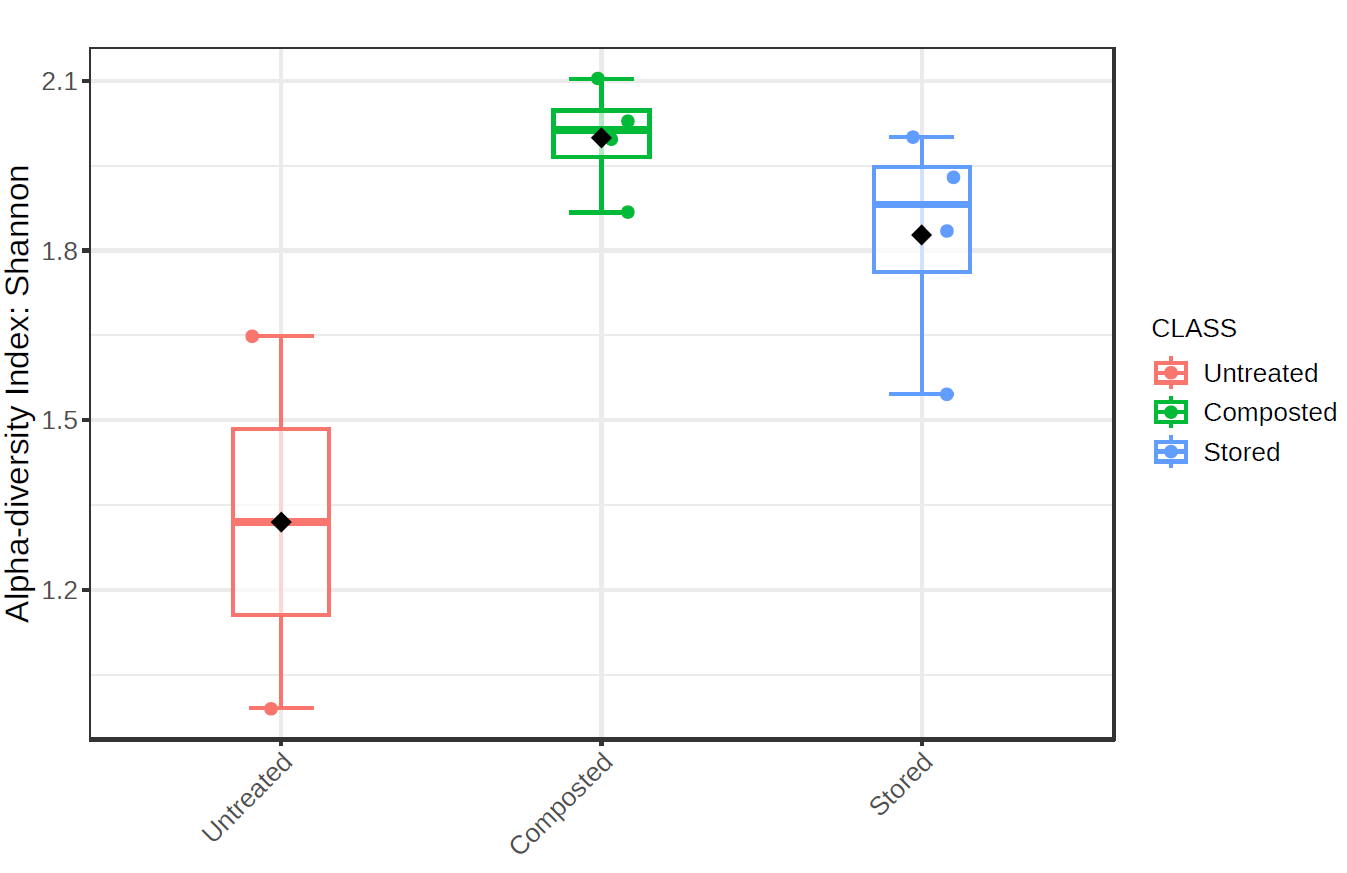


**Figure S2.** Shannon indexes of microbial community diversity (p-value 0.08; Kruskal–Wallis statistic)


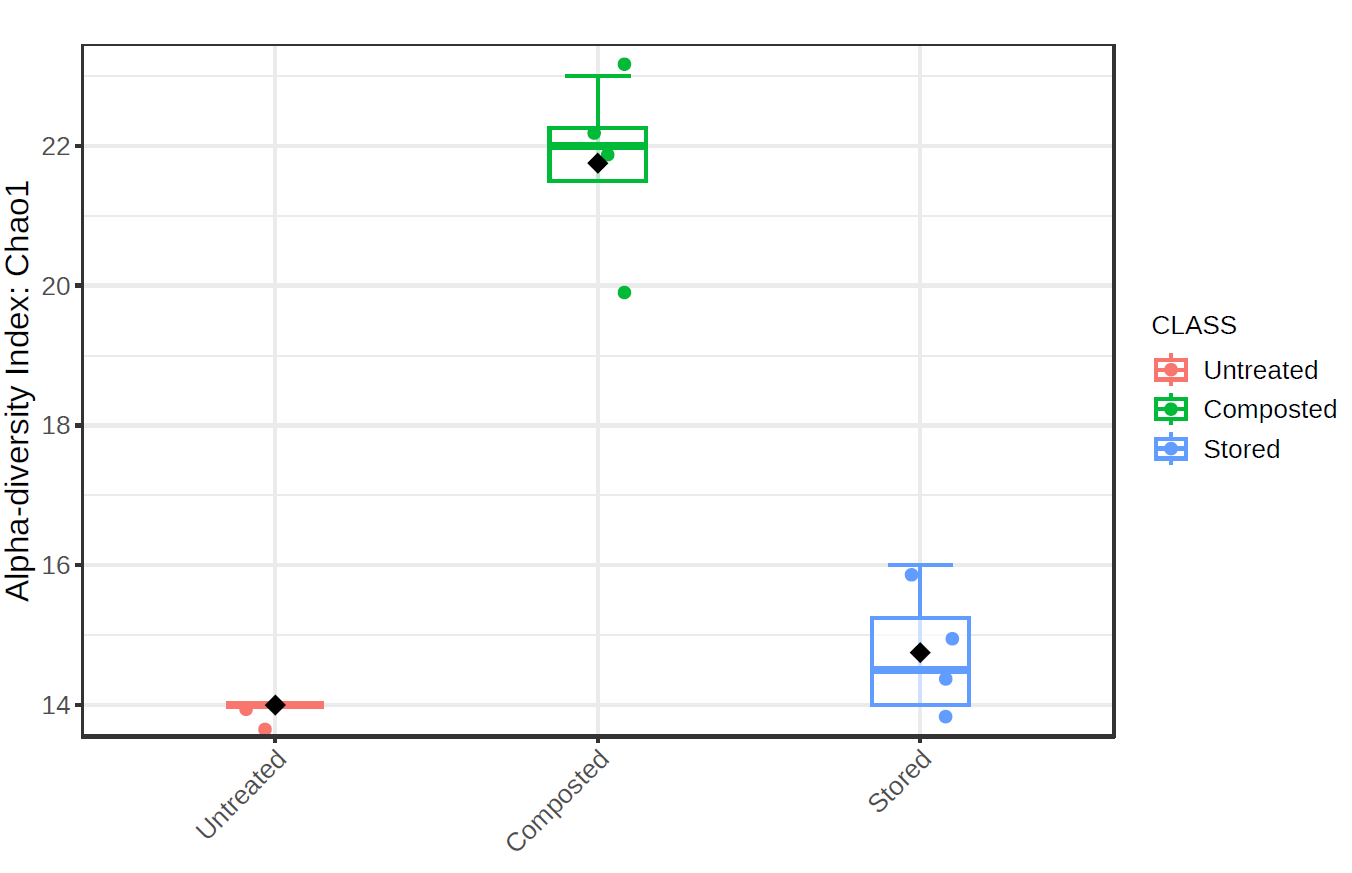


**Figure S3.** Chao1 indexes of microbial community richness (p-value 0.025; Kruskal–Wallis statistic)


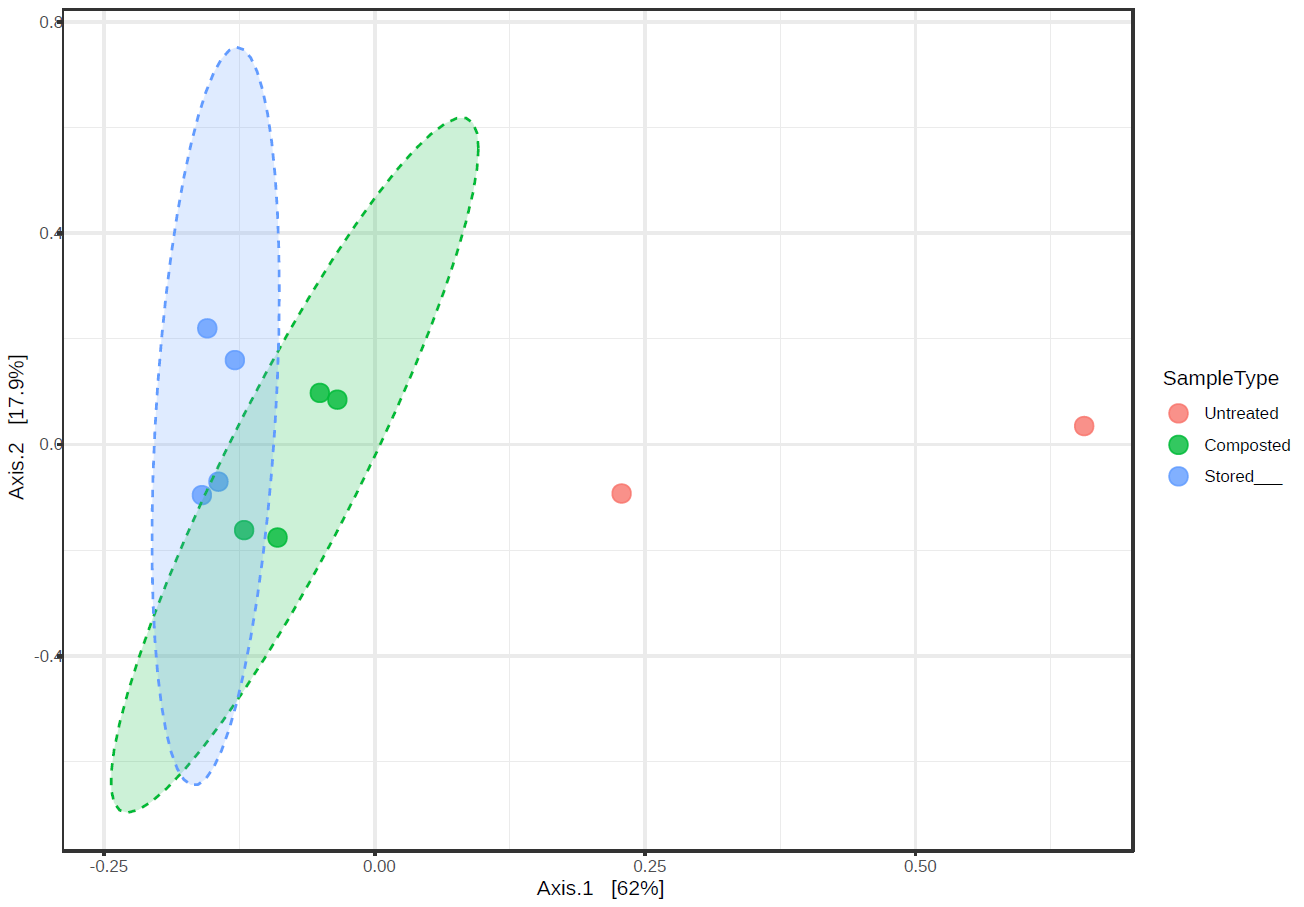


**Figure S4.** The PCoA of taxon abundance in all sample groups, calculated based on the Bray–Curtis distance; [PERMANOVA] F-value: 7.1007; R-squared: 0.66983; p-value: 0.003

**
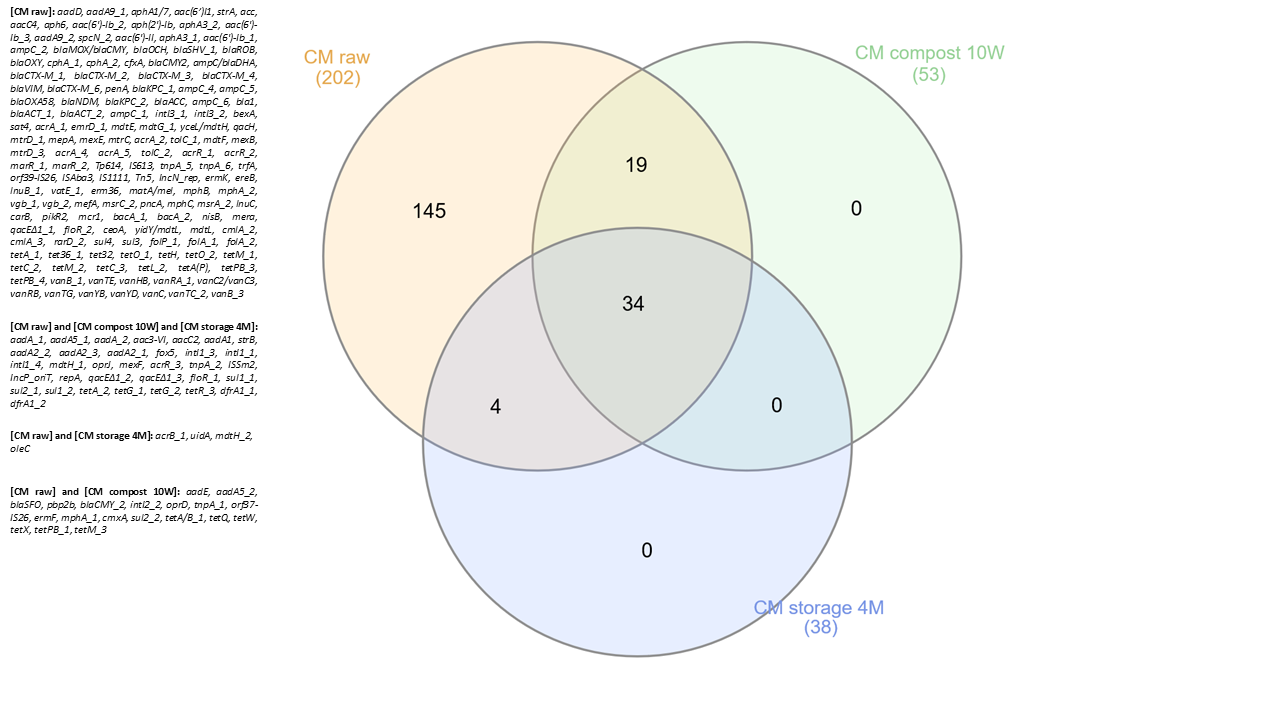
**

**Figure S5.** Comparison between resistomes of untreated cattle manure and resistomes at the end of composting and storage
